# Interaction of Protease-Activated Receptor 2 with G Proteins and β-Arrestin 1 Studied by Bioluminescence Resonance Energy Transfer

**DOI:** 10.3389/fendo.2013.00196

**Published:** 2013-12-20

**Authors:** Mohammed Akli Ayoub, Jean-Philippe Pin

**Affiliations:** ^1^Département de Pharmacologie Moléculaire, Institut de Génomique Fonctionnelle, Montpellier, France; ^2^UMR5203, Centre national de la recherche scientifique, Universités Montpellier 1 & 2, Montpellier, France; ^3^U661, Institut national de la santé et de la recherche médicale, Universités Montpellier 1 & 2, Montpellier, France

**Keywords:** BRET, PAR2, trypsin, G proteins, β-arrestin, pre-assembly

## Abstract

G protein-coupled receptors are well recognized as being able to activate several signaling pathways through the activation of different G proteins as well as other signaling proteins such as β-arrestins. Therefore, understanding how such multiple GPCR-mediated signaling can be integrated constitute an important aspect. Here, we applied bioluminescence resonance energy transfer (BRET) to shed more light on the G protein coupling profile of trypsin receptor, or protease-activated receptor 2 (PAR2), and its interaction with β-arrestin1. Using YFP and Rluc fusion constructs expressed in COS-7 cells, BRET data revealed a pre-assembly of PAR2 with both Gαi1 and Gαo and a rapid and transient activation of these G proteins upon receptor activation. In contrast, no pre-assembly of PAR2 with Gα12 could be detected and their physical association can be measured with a very slow and sustained kinetics similar to that of β-arrestin1 recruitment. These data demonstrate the coupling of PAR2 with Gαi1, Gαo, and Gα12 in COS-7 cells with differences in the kinetics of GPCR-G protein coupling, a parameter that very likely influences the cellular response. Moreover, this further illustrates that pre-assembly or agonist-induced G protein interaction depends on receptor-G protein pairs indicating another level of complexity and regulation of the signaling of GPCR-G protein complexes and its multiplicity.

## Introduction

Recently the study of the interaction of GPCRs with their specific signaling and regulatory proteins has been widely studied using energy transfer-based approaches BRET and FRET (bioluminescence/fluorescence resonance energy transfer) (1–5). These methods allow the measurements, in real-time and live cells, of either the recruitment of specific proteins (i.e., G protein subunits, RGS, arrestins, GRKs …) to the activated receptor or conformational changes within their preassembled or newly formed complexes (6–10). Consequently, interesting conclusions have been reported regarding GPCR-G protein coupling, interaction between G protein subunits, and GPCR-arrestin association in terms of structure, pharmacology, and kinetic of activation/deactivation. Indeed, many studies have shown the possibility of GPCRs to form preassembled complexes even in the absence of receptor activation [for review (1, 3)]. However, others clearly demonstrate that receptor-G protein association is exclusively mediated by agonist activation with expected or unexpected kinetics [for review (1, 3)].

One of the important GPCR families is protease-activated receptors (PARs) which is composed by four subtypes, PAR1, protease-activated receptor 2 (PAR2), PAR3, and PAR4, which play crucial roles in a number of physiological processes such as thrombosis, vascular development, cell proliferation, and tumorigenesis (11). Therefore they are considered as interesting targets for the treatment of various pathologies like inflammation, cancer, and stroke (12). PARs are known to be activated by various serine proteases such as thrombin, trypsin, plasmin, and the factor Xa (13, 14). PARs activation occurs through a highly specific protease-mediated cleavage of the N-terminal extremity of the receptor unmasking a new N terminus that acts as a tethered ligand which directly activates the transmembrane core of the cleaved receptor (13–15). PARs are characterized by the diversity and overlapping of their signaling pathways involving various G protein classes: Gαi/o, Gαq/11, Gα12/13 proteins as well as arrestins promoting multiple downstream signaling responses in various cellular models (15–19). Furthermore, PARs undergo a rapid desensitization, internalization, and degradation involving the phosphorylation of the receptor by G protein-coupled receptor kinases and the recruitment of arrestins (18, 19). However, except for the prototype member, PAR1, the G protein coupling profile of the different members of PARs is not really a consensus matter and little information is available regarding their coupling to G proteins. This is true for PAR2 which is typically Gαq/11-coupled receptor leading to an increase in intracellular calcium via PLC/IP3 pathway (16, 18, 20, 21). However, a study in the Xenopus oocyte system has reported that PAR2-mediated intracellular signaling events were a pertussis toxin (PTX)-sensitive indicating a role of PAR-Gαi/Gαo coupling (22). Also, PAR2 activation in the epithelial cells elicited a calcium response in both PTX-sensitive and PTX-insensitive depending on the cell model used (23). Recently PAR2 activating peptide SLIGRL has been shown to induce smooth muscle contraction by triggering the activation of Gαq, Gαi1, and Gα13 (24). However, it has been shown that PAR2, in contrast to PAR1, does not couple to Gαi and Gαo families in COS-7 cells (25). Together, these studies indicate that the pattern of G protein coupling of PAR2 strongly depends on the cellular model considered since the differences can be due to factors such receptor density, the availability of G proteins and other interacting proteins … etc. [For review (1)].

Many recent studies have used BRET to investigate GPCR-G protein coupling (1, 3) including PARs (6, 7, 26). Indeed, our recent data using both BRET and time-resolved FRET (TR-FRET) technologies revealed the existence of preassembled complexes between PAR1 and Gαi1 protein (6, 7), as well as Gαo (27) in COS-7 cells. In contrast, the physical association of PAR1 with Gα12, but not Gα13, was exclusively observed upon receptor activation with a very slow and stable kinetic indicating the recruitment of Gα12 to the activated PAR1 in parallel to β-arrestin1 recruitment (7). In this study, we aimed to investigate the physical interaction of PAR2 with Gαi1, Gαo, Gα12, and β-arrestin1 before and upon receptor activation by BRET, in real-time and live cells, using Rluc-tagged Gα proteins and YFP-tagged PAR2.

## Materials and Methods

### Materials and plasmid constructions

Human cDNA for PAR2 were cloned into pcDNA3.1+ (Guthrie Research Institute, Sayre, PA, USA). PAR2-YFP fusion protein and Rluc-tagged G proteins were generated as previously described (6). PAR2-ΔC-YFP mutant corresponds to the δ Tail mutant reported by Seatter et al. removing the entire C-terminus from Serine 348 (28). Such truncation was generated using the similar strategy for PAR1-ΔC-YFP previously reported (6). Rluc-β-arrestin1 were generously provided by M. G. Scott (Institut Cochin, Paris, France). Bovine trypsin pancreas was from Calbiochem Merck KgaA (Darmstadt, Germany) and Ser-Leu-Iso-Gly-Arg-Leu-NH2 (SLIGRL) peptide was from Tocris Cookson Inc., Ellisville, MO, USA. Ninety-six-well white microplates were from Greiner Bio-One SAS (Courtaboeuf, France). Coelenterazine h substrate was from Promega (Charbonnières, France).

### Cell culture and transfection

COS-7 cells were grown in complete medium [DMEM supplemented with 10% (v/v) fetal bovine serum, 4.5 g/l glucose, 100 U/ml penicillin, 0.1 mg/ml streptomycin, and 1 mM glutamine] (all from Invitrogen, Carlsbad, CA, USA). Transient transfections were performed by reverse transfection in 96-well plate using Lipofectamine 2000 following the manufacturer’s protocol. Briefly, for each well the different combinations of coding plasmids were used as follow: 25 ng of PAR2-YFP (WT and ΔC mutant), 50 ng of Gαi1/o-Rluc or Rluc-β-arrestin 1, and 150 ng of Gα12-Rluc. The plasmid mixes and Lipofectamine 2000 (0.5 μl/well) were first preincubated 5 min at room temperature in serum-free DMEM (2 × 25 μl/well). Then the two solutions of serum-free DMEM containing plasmids and Lipofectamine were mixed and incubated 20 min at room temperature. Cells (10^5^ in 150 μl/well) in DMEM supplemented with 10% FCS were then incubated with the final plasmid-Lipofectamine mix (50 μl/well).

### BRET measurements

Forty-eight hours after transfection cells were washed with PBS and preincubated in the absence or presence of trypsin or SLIGRL as indicated in PBS at 37°C. Cells were then washed and resuspended in PBS for BRET measurements. The kinetic and dose-response analysis of BRET signals was performed as described previously (6). The BRET Ratio was defined as the subtraction of the ratio of the emission at 530 ± 25 nm over the emission at 485 ± 20 nm of cells expressing the Rluc fusion protein alone from the same ratio of cells co-expressing Rluc and YFP fusion proteins. Then the resulted values were multiplied by 1000. However, the ligand-induced BRET was calculated by subtracting the BRET Ratio for a PBS-treated cell sample from the same ratio for the aliquot of the same cells treated with agonist. In this calculation only ligand-promoted BRET changes are represented and the PBS-treated cell sample represents the background eliminating the requirement for measuring an Rluc-only control sample especially when fast kinetics and dose-response analysis are performed.

### Data analysis

All data were represented using Prism GraphPad software (San Diego, CA, USA). Kinetic and dose-response curves were fitted with non-linear regression equations using the different equations as indicated.

## Results

### Basal and ligand-induced BRET between PAR2 and Gα proteins

**Figure 1:**
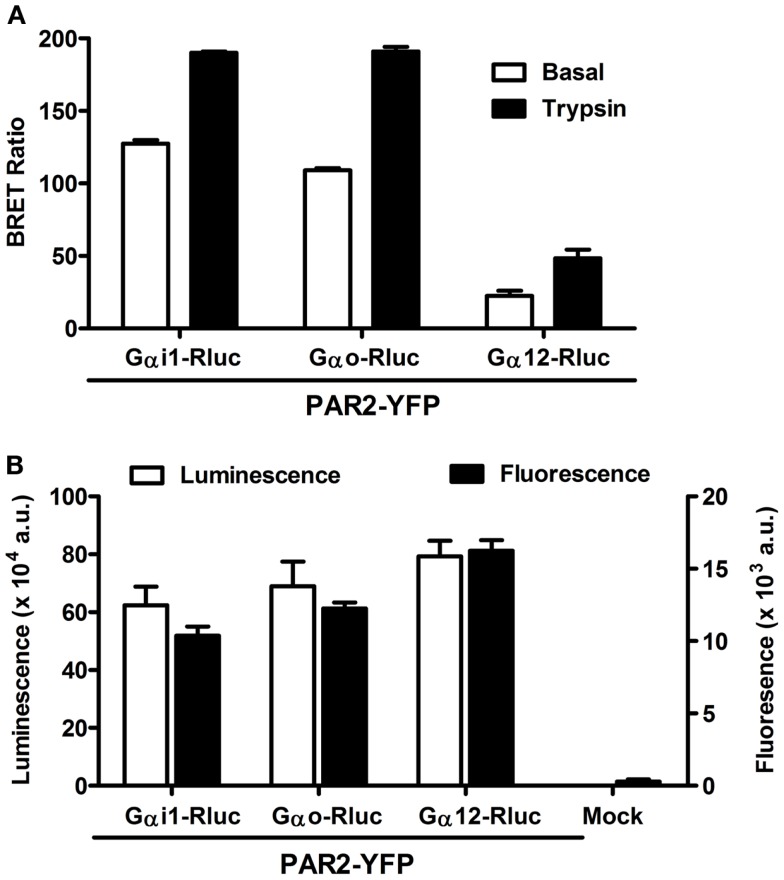
**Bioluminescence resonance energy transfer between PAR2 and Gα proteins in live COS-7 cells**. **(A)** BRET measurements in COS-7 cells co-expressing PAR2-YFP and either Gαi1-Rluc, Gαo-Rluc, or Gα12-Rluc in the absence (□) and presence of stimulation with 100 nM of trypsin (■) for 2 min (for Gαi and Gαo) or 30 min for (Gα12). **(B)** Quantification of the luciferase (Rluc) activity (□) and YFP fluorescence (■) of BRET partners measured in BRET assay. Data are means ± SEM of three independent experiments performed in triplicate.

The pattern of G protein coupling for PAR2 is still not completely clarified when compared to PAR1 which is known to activate Gαo, Gαi1/2, Gαq as well as Gα12/13 pathways in various *in vitro* and *in vivo* models (14, 29). Therefore, we wanted to investigate the putative coupling of PAR2 with Gαi1, Gαo, and Gα12, as this has been previously demonstrated for PAR1 (6, 7, 27). For this, we used BRET approach allowing real-time assessment of the receptor-G protein complexes in live cells and BRET measurements were performed in COS-7 cells transiently co-expressing Gα-Rluc and PAR2-YFP fusion proteins and stimulated or not with its specific agonist, trypsin. As shown in Figure 1A, significant constitutive BRET signal was measured between PAR2-YFP and either Gαi1-Rluc or Gαo-Rluc compared to Gα12-Rluc. This was observed at similar relative expression levels of PAR2-YFP as well as Rluc-tagged G proteins measured by fluorescence and luminescence, respectively (Figure 1B). Interestingly, the stimulation with 100 nM of trypsin for 2 min (for Gαi and Gαo) or 30 min for (Gα12) specifically increased the BRET signal between all the Gα-Rluc and PAR2-YFP indicating functional coupling of PAR2 with Gαi1, Gαo, and Gα12 (Figure 1A). Together, these data suggest a possible pre-assembly between PAR2 and Gαi1 and Gαo, but not Gα12. The agonist-induced BRET increase clearly demonstrates a functional coupling of PAR2 with these G proteins which is characterized by conformational changes within the preassembled PAR2-Gαi1 and PAR2-Gαo complexes and probably Gα12 recruitment as previously shown for PAR1 (6, 7, 27).

### Kinetic analysis of ligand-induced BRET between PAR2 and Gα proteins

**Figure 2:**
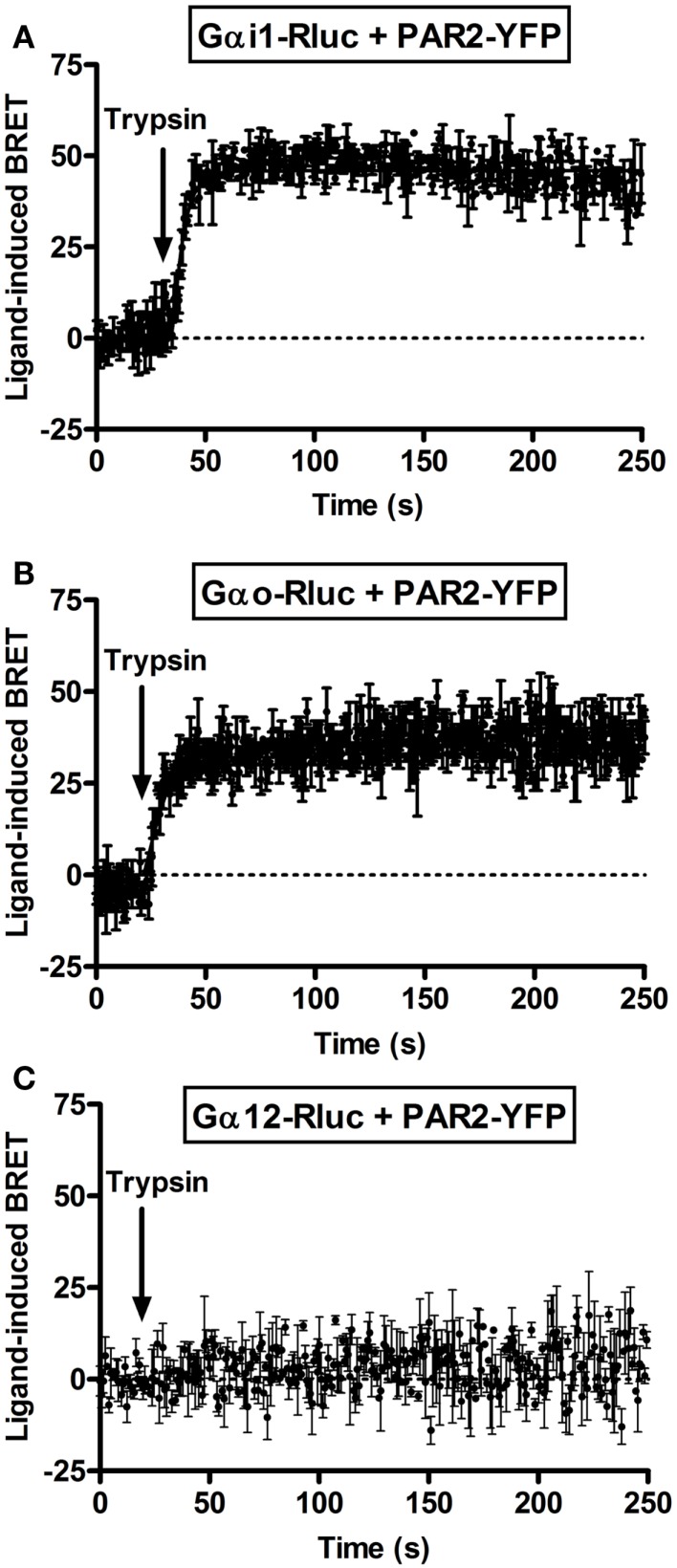
**Kinetic analysis of ligand-induced BRET increase between PAR2 and Gα proteins**. COS-7 cells transiently co-expressing PAR2-YFP and either Gαi1-Rluc **(A)**, Gαo-Rluc **(B)**, or Gα12-Rluc **(C)** were used for BRET experiments and repetitive signals were recorded before and immediately after the injection of 100 nM of trypsin. The curves were fitted using “Plateau then increase to top” equation of Prism GraphPad software and *Y* = IF[*X* < *X*0, Plateau, Plateau + (Top − Plateau)*(1 − exp(−K* (*X−X*0)))] constraining the plateau to a theoretical value of 0. Data are mean ± SEM of three independent experiments performed in single points.

Next, we performed real-time kinetics before and after agonist addition using the injection system available on the Mithras LB-490. As result, the injection of 100 nM of trypsin rapidly increased the BRET signal between PAR2-YFP and Gαi1-Rluc (Figure 2A) as well as Gαo-Rluc (Figure 2B) and the increased signal remained stable ~5 min after ligand injection. The *t*_1/2_ values are in second interval as indicated in Table 1. However, no ligand-induced BRET increase was observed between PAR2-YFP and Gα12-Rluc within the first 4 min post-stimulation (Figure 2C). These observations are comparable to what we previously reported on PAR1-Gαi1 coupling (6, 7) indicating similar pre-assembly properties and activation kinetics.

**Table 1:** ***t*_1/2_ Values of trypsin-induced BRET increase signals and its decline**.

BRET combinations	BRET increase	BRET decline
Gαi1-Rluc + PAR2-YFP	3.31 ± 0.81 s	9.82 ± 0.38 min
Gαo-Rluc + PAR2-YFP	1.80 ± 0.40 s	9.96 ± 0.57 min
Gα12-Rluc + PAR2-YFP	4.94 ± 0.53 min	ND
Rluc-β-arrestin 1 + PAR2-YFP	1.72 ± 0.29 min	ND
	3.29 ± 0.04 min^a^	

*^a^*t*_1/2_ Value for SLIGRL. Data are mean ± SEM (*n* = 3)*.

**Figure 3:**
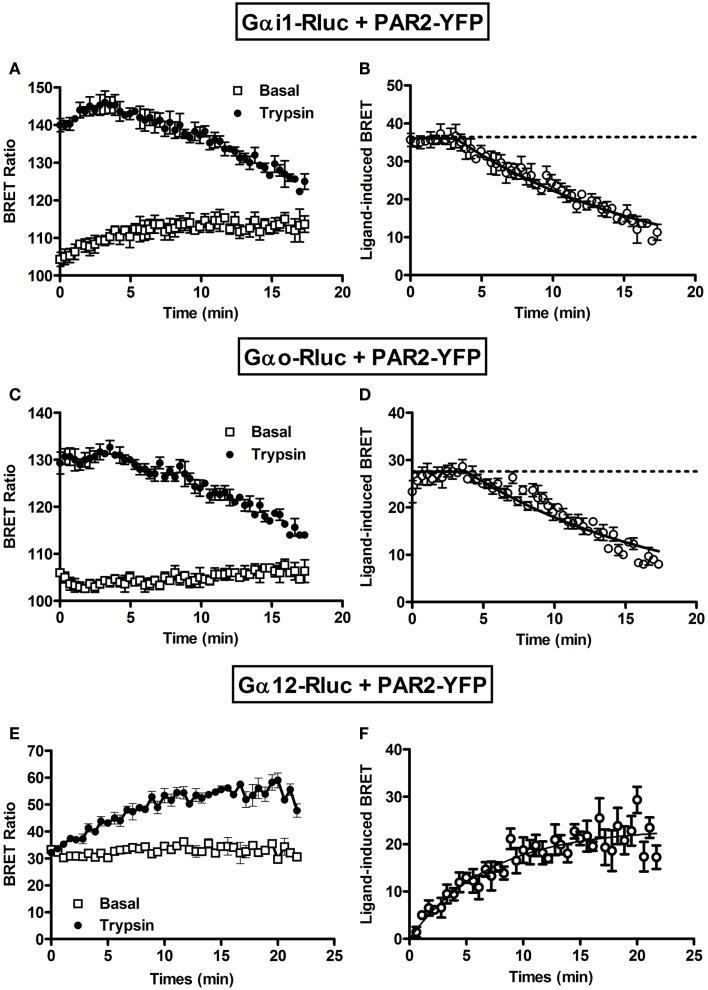
**Long-term kinetic analysis of trypsin-induced BRET increase between PAR2 and Gα proteins**. COS-7 cells transiently co-expressing PAR2-YFP and either Gαi1-Rluc **(A,B)** or Gαo-Rluc **(C,D)** or Gα12-Rluc **(E,F)** were used for BRET experiments in the absence (□) or presence of 100 nM of trypsin (●). For this, repetitive signals were recorded every ~35 s immediately after trypsin addition during 18–20 min. The panels **(B,D,F)** represent the Prism GraphPad fits of trypsin-induced BRET increase using either “Plateau followed by one phase decay” equation (*Y* = IF[*X* < *X*0, *Y*0, Plateau + (*Y*0 − Plateau)*exp(−K*(*X−X*0))]) constraining the plateau to a theoretical value of 0 for Gαi1 and Gαo or “One phase exponential association” equation (*Y* = *Y*_max_*(1−exp(−K*X)) for Gα12. Data are mean ± SEM of three independent experiments performed in single points.

Next, we performed long-term kinetics (up to 15–20 min) in the absence or presence of trypsin stimulation. As shown above, for both Gαi1-Rluc (Figure 3A) and Gαo-Rluc (Figure 3C) we observed a basal BRET signal and trypsin promoted a rapid BRET increase in the first seconds of stimulation and the signal was stable for ~4 min before its slow decline in a time-dependent manner. The kinetic analysis using “Plateau followed by one phase decay” equation of Prism GraphPad software resulted in decay *t*_1/2_ values close to 10 min (Table 1) for both Gαi1-Rluc (Figure 3B) and Gαo-Rluc (Figure 3D). This analysis demonstrates a reversible trypsin-induced BRET increase reflecting a rapid activation of PAR2-Gαi1 and PAR2-Gαo complexes which is then likely followed by their desensitization. In contrast, a very low BRET signal was measured between Gα12-Rluc and PAR2-YFP as expected (Figure 3E) consistent with the data in Figure 1A. Interestingly, in the presence of trypsin we observed a gradual increase in the BRET signal between Gα12-Rluc and PAR2-YFP (Figure 3E) which reached a plateau after 15 min of stimulation (Figure 3F) with a *t*_1/2_ value close to 5 min (Table 1).

Together, our data indicate a pre-assembly of PAR2 with Gαi1 and Gαo but not Gα12 and nicely demonstrate the rapid agonist-promoted activation of the preassembled PAR2-G protein complexes. For Gαi1 and Gαo BRET increase likely reflects conformational changes within the preassembled complexes leading to their activation followed by their time-dependent desensitization. In contrast, the kinetic data with Gα12 suggest a delayed recruitment in time-dependent manner of the G protein to the activated PAR2. All these observations are in fact consistent with our previous data on PAR1-Gαi1 coupling (6, 7) suggesting similar profile and properties with regard to G protein coupling.

### Dose-response analysis of ligand-induced BRET increase between PAR2 and Gα proteins

**Figure 4:**
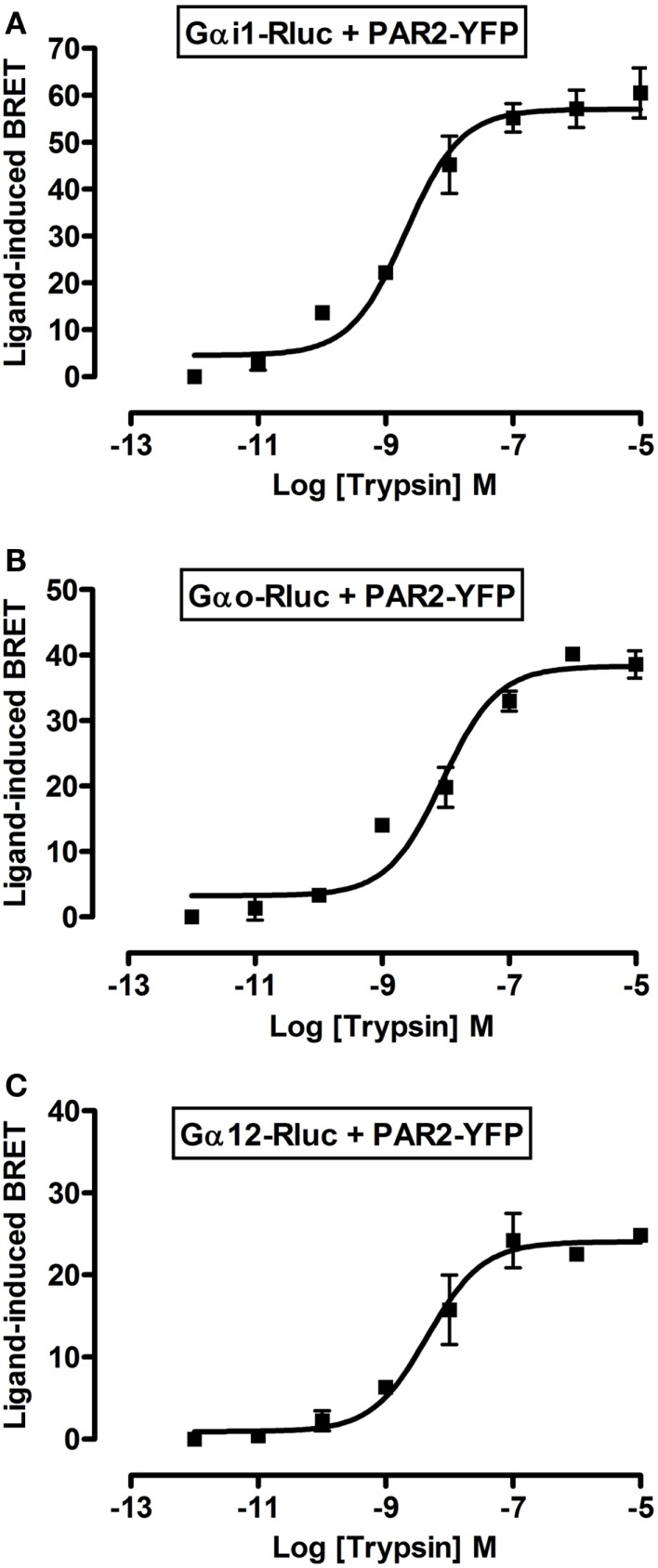
**Dose-response analysis of trypsin-induced BRET increase between PAR2 and Gα proteins**. COS-7 cells transiently co-expressing PAR2-YFP and either Gαi1-Rluc **(A)**, Gαo-Rluc **(B)**, or Gα12-Rluc **(C)** were used for BRET experiments in the presence of increasing concentrations of trypsin as indicated. Data are means ± SEM of three independent experiments performed in duplicate.

**Figure 5:**
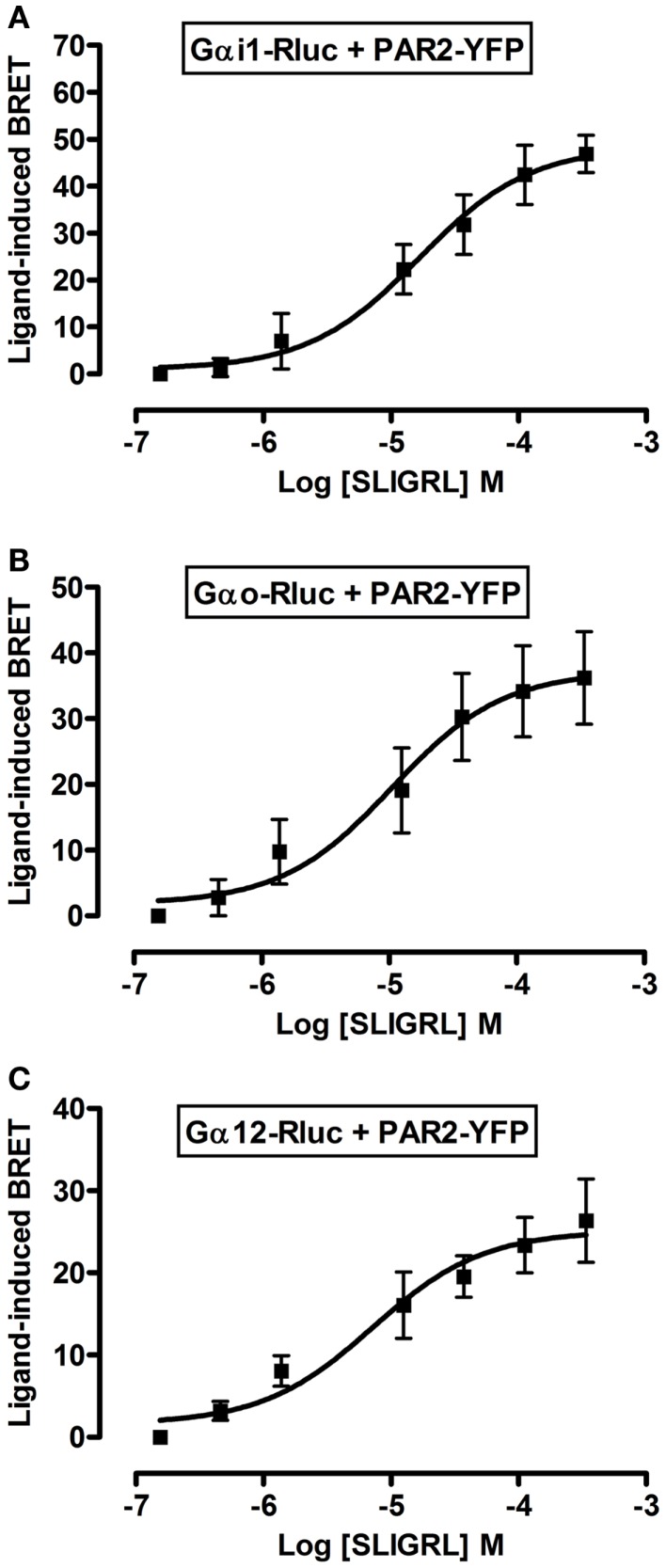
**Dose-response analysis of SLIGRL-induced BRET increase between PAR2 and Gα proteins**. COS-7 cells transiently co-expressing PAR2-YFP and either Gαi1-Rluc **(A)**, Gαo-Rluc **(B)**, or Gα12-Rluc **(C)** were used for BRET experiments in the presence of increasing concentrations of SLIGRL as indicated. Data are means ± SEM of three to four independent experiments performed in duplicate.

To further profile PAR2-G protein interactions and demonstrate the specificity of ligand-induced BRET increase between Rluc-tagged Gα and PAR2-YFP being associated to the activation of receptor-G protein complex we carried out dose-response analysis. After stimulation of cells with increasing doses of trypsin according to the kinetic profile of Gαi1-Rluc, Gαo-Rluc, and Gα12-Rluc, shown in Figure 3, a significant BRET increase was measured in a dose-dependent manner for Gαi1-Rluc/PAR2-YFP (Figure 4A), Gαo-Rluc/PAR2-YFP (Figure 4B), or Gα12-Rluc/PAR2-YFP (Figure 4C) complexes. To further demonstrate the specificity of trypsin effects, we also performed dose-response experiments using PAR2-selective peptide agonist, SLIGRL, which does not require receptor cleavage to activate PAR2 (20). As shown in Figure 5, SLIRGL also induced a significant BRET increase was measured in a dose-dependent manner between Gαi1-Rluc (Figure 5A), Gαo-Rluc (Figure 5B), or Gα12-Rluc (Figure 5C) and PAR2-YFP. Both trypsin and SLIGRL increased BRET signals with their expected and respective potencies (20) consistent with ligand-induced BRET increase being reflecting PAR2-G protein complex activation (Table 2).

**Table 2:** **pEC_50_ values of trypsin and SLIGRL on BRET signals**.

BRET combinations	Trypsin	SLIGRL
Gαi1-Rluc + PAR2-YFP	8.61 ± 0.08 (*n* = 3)	4.86 ± 0.31 (*n* = 4)
Gαo-Rluc + PAR2-YFP	8.03 ± 0.18 (*n* = 3)	5.18 ± 0.26 (*n* = 4)
Gα12-Rluc + PAR2-YFP	8.31 ± 0.29 (*n* = 3)	5.14 ± 0.37 (*n* = 3)
Rluc-β-arrestin 1 + PAR2-YFP	7.86 ± 0.11 (*n* = 3)	4.91 ± 0.07 (*n* = 3)

*Data are mean ± SEM (*n* = 3–4)*.

### Ligand-induced recruitment of β-arrestin 1 to PAR2

**Figure 6:**
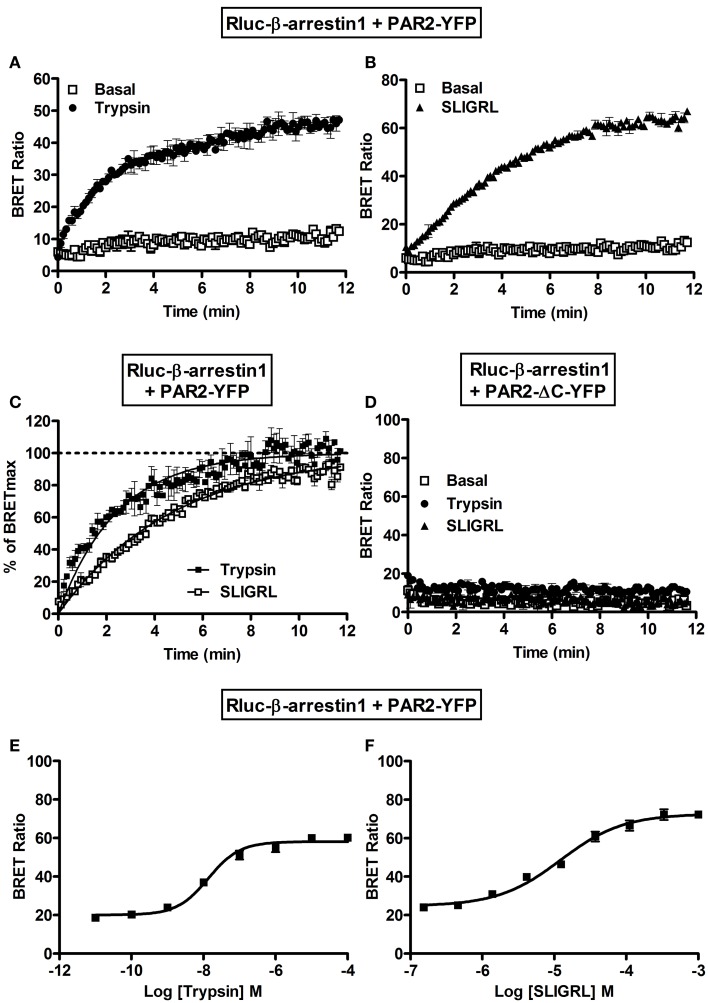
**Recruitment of β-arrestin 1 to the activated PAR2 studied by BRET**. Time-course analysis on BRET signals measured in COS-7 cells transiently co-expressing Rluc-β-arrestin 1 and either PAR2-YFP **(A,B,C)** or PAR2-ΔC-YFP **(D)** in the absence (□) or presence of 100 nM of trypsin (●) or 10 μM of SLIGRL (Δ). **(C)** Represents the normalization of the curves in **(A,B)** together on the percentage of the maximal ligand-induced BRET signals and the curves were fitted by Prism GraphPad fits of trypsin-induced BRET increase using “One phase exponential association” equation (*Y* = *Y*_max_*(1−exp(−K*X))). Dose-response analysis on the increase of BRET signals between Rluc-β-arrestin 1 and PAR2-YFP upon stimulation with increasing concentrations of trypsin **(E)** or SLIGRL **(F)** as indicated. Data are means ± SEM of three independent experiments performed in duplicate.

Finally, we examined the interaction of PAR2 with β-arrestin 1 using BRET. Indeed, the activation of PAR2 is known to be followed by its desensitization and phosphorylation at multiple serine/threonine residues in the C-terminal tail (18, 28, 30). Such phosphorylation constitutes a key step for β-arrestin recruitment to PAR2 promoting receptor internalization through clathrin-coated pits (30). As expected no significant basal BRET can be measured Rluc-β-arrestin 1 and PAR2-YFP and both 100 nM of trypsin (Figure 6A) and 10 μM SLIGRL (Figure 6B) nicely increased BRET signals. The BRET increase was time-dependent before it reached a plateau corresponding to a saturation of all the phosphorylated PAR2 with the recruited β-arrestin 1. After normalization of the data in Figures 6A and 6B to the percentage of maximal BRET in each case we noticed a slight shift in the kinetics between trypsin and SLIGRL curves (Figure 6C) with the *t*_1/2_ values indicated in Table 1. This difference in the kinetics may be due to differences in the binding and activation properties of trypsin and SLIGRL. To demonstrate the specificity of the ligand-induced BRET increase as well as the requirement of PAR2 phosphorylation for β-arrestin 1 recruitment, we used a mutant of PAR2 (PAR2-ΔC-YFP) lacking a large part of its C-terminus (from serine 348) containing multiple serine/threonine residues (28). As shown in Figure 6D, the deletion of PAR2 C-terminus completely abolished the ligand-promoted BRET increase demonstrating its implication in PAR2-β-arrestin 1 association. Moreover, both trypsin (Figure 6E) and SLIGRL (Figure 6F) induced β-arrestin 1 recruitment to PAR2 in a dose-dependent manner with similar potencies (Table 2). These BRET observations clearly show a recruitment of β-arrestin 1 to PAR2 involving the C-terminus of the receptor as previously shown (28).

## Discussion

In this study we investigated the interaction of PAR2 with three different G protein subunits, Gαi1, Gαo, and Gα12 as well as β-arrestin1 in live COS-7 cells and in real-time using BRET. We demonstrated the existence of preassembled PAR2-Gαi1 and PAR2-Gαo complexes which are nicely activated by trypsin and SLIGRL (PAR2-selective peptide agonist) indicating the coupling of PAR2 to Gαi1 and Gαo proteins in our model. However, the association of PAR2 with Gα12 protein was exclusively observed upon receptor activation similarly to β-arrestin1 recruitment suggesting different coupling mode of PAR2 with Gα12. The dose-response analysis indicated the activation of PAR2-G protein complexes with the known potencies of both trypsin and SLIGRL (20). Together, these findings are similar to what we previously reported on thrombin receptor (PAR1) (6, 7, 27) as well as other studies with other GPCR-G protein pairs (8, 31, 32).

The kinetic analysis showed that PAR2 activation led to a rapid and transient BRET increase between the receptor and either Gαi1 or Gαo proteins with *t*_1/2_ values fluctuating from 1 to 4 s. Such BRET increase likely reflects conformational changes within the activated preassembled complexes as shown for PAR1 (6, 7). Also, the rapid activation of the preassembled complexes is rather slower but still consistent with the activation kinetics observed with other GPCRs (33–35). This is in agreement with the fast kinetic for the activation of these classes of G proteins leading to rapid modulation of intracellular cAMP levels. In fact, such GPCR-G protein pre-assembly has been reported to be important to favor a certain GPCR-G protein stoichiometry required for rapid and targeted downstream cellular responses (36).

Moreover, long-term kinetic analysis revealed that the rapid agonist-induced activation of PAR2-Gαi/Gαo complexes is followed by the desensitization in time-dependent manner of the preassembled complexes. These observations are supported by the assessment of β-arrestin 1 recruitment to the activated PAR2 which showed a time-dependent association between PAR2 and β-arrestin 1 upon receptor activation with either trypsin or SLIGRL. Moreover, we further demonstrated the importance of PAR2 C-terminus for such interaction as previously reported (18, 30).

For the interaction with Gα12 protein, the data with PAR2 support our previous data with PAR1 (7). This G protein seems to be recruited to PAR2 according to an agonist-dependent process with recruitment kinetics similar to that of β-arrestin 1 (Table 1). This kinetics may be reconciled with the kinetics the activation of the small G protein RhoA and p115RhoGEF, two major protein effectors of G12/13 family (37, 38), as well as the involvement of G12/13 in slow and long-term cellular responses such as proliferation, differentiation, and migration (39, 40). However, our previous study clearly indicated that slow and sustained Gα12 recruitment cannot be considered general to all GPCRs since its pre-assembly has been demonstrated with other GPCRs (7). Therefore, whether such pre-assembly with Gαi1/o versus agonist-dependent Gα12 recruitment constitute a general feature of protease-activated receptor family or rather reflect similarities in G protein coupling between PAR1 and PAR2 this needs further investigations. Our study demonstrating the functional interaction of PAR2 with Gαi1, Gαo, and Gα12 in COS-7 cells, in a similar way to PAR1 (6, 7) shed more light on the G protein coupling of PAR2. Our observations are in agreement with the previous studies showing the coupling of PAR2 to Gαi1 in the rabbit gastric muscle cells (24) as well as PAR2 forming a stable complex with Gα12 in COS-7 cells (25). However, the latter also reported that PAR2 did not activate Gαi1- and Gαo-dependent signaling pathways (25) illustrating the complexity of PAR2-G protein coupling which appears to be strongly dependent on the cellular model considered.

Finally, our study further illustrates that the GPCR-G protein pre-assembly and agonist-dependent G protein recruitment depend on the receptor-G protein pair and the cellular background of the model used. This may constitute an important level of integration and regulation of the multiple coupling of GPCRs (1, 41–43), especially when considering the new concepts of GPCR biased signaling and heteromerization.

## Conflict of Interest Statement

The authors declare that the research was conducted in the absence of any commercial or financial relationships that could be construed as a potential conflict of interest.
